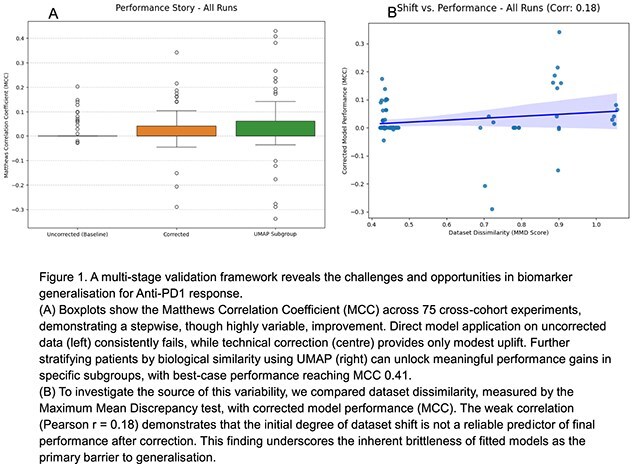# Deconstructing biomarker generalisation failure in anti-pd1 cancer immunotherapy response: a multi-cohort framework

**DOI:** 10.1093/bib/bbaf631.010

**Published:** 2025-12-12

**Authors:** Elizabeth Amelia, Chayanit Piyawajanusorn, Pedro Ballester

**Affiliations:** Department of Bioengineering, Imperial College London, United Kingdom; HRH Princess Chulabhorn College of Medical Sciences, Thailand; Department of Bioengineering, Imperial College London, United Kingdom

## Abstract

**Introduction:**

Predictive biomarkers are central to precision oncology [1], yet transcriptomic signatures for predicting response to Anti-PD1 immunotherapy rarely generalise beyond their discovery cohort. This persistent failure, often attributed to dataset shift, remains poorly understand and hampers clinical translation. We sought to move beyond simple validation and systematically examine the causes of generalisation failure in melanoma and renal cell carcinoma, hypothesising that the brittleness of fitted models is a key barrier.

**Methods:**

We assembled five independent transcriptomic cohorts of melanoma and renal cell carcinoma patients treated with Anti-PD1 therapy. Four cohorts (N = 360) were designated as discovery sets from which we generated 20 distinct gene-panel biomarkers using our BioAdapt pipeline. This consensus framework [2] repeatedly partitions data with different random seeds, applies feature selection, and fits logistic regression models with optimised thresholds, ensuring variability is captured rather than relying on a single split. For validation, each model trained on one cohort was systematically applied to the remaining independent datasets, producing 75 fully cross-cohort experiments where no model was ever tested on its own training data. To understand generalisation failure, we designed a three-stage framework: (1) **Diagnosis** of dataset dissimilarity using the Maximum Mean Discrepancy test (a statistical test of distributional shift), (2) **Technical correction** with label-free quantile normalisation, and (3) **Biological stratification** with UMAP to identify subgroups most similar to the training cohort.

**Results:**

Direct model transfer failed, yielding near-random performance (mean MCC 0.016). For clinicians, this means that a biomarker trained on one hospital’s patients is very unlikely to work in another without adaptation. Diagnostic testing confirmed pervasive dataset shift (mean Maximum Mean Discrepancy 0.584). Correction produced only modest gains (mean corrected MCC 0.026). However, stratification revealed patient subgroups where performance nearly doubled (best-case MCC 0.41), demonstrating that predictive utility is often local to biologically defined subsets. In practice, this suggests biomarkers may still be valuable if applied to the right subset of patients, rather than universally.

**Discussion & Conclusion:**

Our analysis shows that generalisation failure stems less from technical batch effects than from the intrinsic brittleness of fitted models. While correction is necessary, stratifying patients by biological similarity can recover predictive signal in defined contexts. These findings quantify the limits of current biomarker models and highlight the need for inherently robust, transferable approaches such as transfer learning or domain adaptation to achieve clinical translation. Importantly, this work provides a realistic roadmap for the field by showing where predictive biomarkers are most likely to succeed and in which patient populations they can be deployed with confidence. By reframing validation from a simple pass or fail exercise to a deeper diagnostic process, our framework offers a translationally relevant pathway towards biomarker deployment in clinical trials, ultimately enabling the stratification of patients who are most likely to respond to Anti-PD1 therapy and accelerating precision immuno-oncology. For clinicians, this means moving towards a future where biomarker-guided patient selection for Anti-PD1 treatment is both feasible and reliable, reducing the risk of exposing non-responders to ineffective therapies.

**References:**

1. Piyawajanusorn C., Ghislat G., Ballester P.J. ‘Predicting atezolizumab response in metastatic urothelial carcinoma patients using machine learning on integrated tumour gene expression and clinical data.’ NPJ Precis Oncol. 2025;9(1):170.

2. Abeel T., Helleputte T., Van de Peer Y., Dupont P., Saeys Y. ‘Robust biomarker identification for cancer diagnosis with ensemble feature selection methods.’ Bioinformatics. 2010;26(3):392–398.